# Data on proteins of lysenin family in coelomocytes of *Eisenia andrei* and *E. fetida* obtained by tandem mass spectrometry coupled with liquid chromatography

**DOI:** 10.1016/j.dib.2016.09.035

**Published:** 2016-09-29

**Authors:** Bianka Swiderska, Sylwia Kedracka-Krok, Barbara Plytycz

**Affiliations:** a Department of Physical Biochemistry, Faculty of Biochemistry, Biophysics and Biotechnology, Jagiellonian University, Kraków, Poland; b Laboratory of Proteomics and Mass Spectrometry, Malopolska Centre of Biotechnology, Jagiellonian University, Krakow, Poland; c Department of Evolutionary Immunology, Institute of Zoology, Faculty of Biology and Earth Sciences, Jagiellonian University, Kraków, Poland

**Keywords:** *Eisenia fetida*, *Eisenia andrei*, Lysenin-related proteins, LPRs, Mass spectrometry, LC-MS/MS

## Abstract

The data described are related to the article “Lysenin family proteins in earthworm coelomocytes – comparative approach” (B. Swiderska, S. Kedracka-Krok, T. Panz, A.J. Morgan, A. Falniowski, P.Grzmil, B. Plytycz, 2016) [1]. Lysenin family proteins were identified based on unique peptides sequenced by tandem mass spectrometry coupled with liquid chromatography (LC-MS/MS) in lumbricid earthworms *Eisenia andrei* and *E. fetida*, the latter with or without the MUG-like fluorophore. Lysenin and lysenin-related protein 2 (LRP-2, fetidin) were identified in all 9 investigated specimens of *Eisenia* sp. LRP-1 was identified in 5 of 6 specimens of *E. fetida*, while LRP-3 was present in 2 of 3 investigated specimens of *E. andrei*. Here, the detailed characteristics of identified peptides unique to the particular members of lysenin family present in each particular earthworm specimen was provided. The information concerning mass to charge ratio, retention time, modifications and score of unique peptides was given.

**Specifications Table**

**Table:** 

Subject area	Biology
More specific subject area	Comparative immunology, proteomics
Type of data	Tables
How data was acquired	LC-MS/MS measurements were performed with Q-Exactive mass spectrometer (Thermo Fisher Scientific, Bremen, Germany) equipped with a DPV-550 Digital PicoView nanospray source (New Objective, USA) and coupled with an UltiMate 3000RS LC nanoSystem (Dionex, Thermo Fisher Scientific, USA), raw data analysis was done with Proteome Discoverer 1.4 (Thermo Fisher Scientific, USA).
Data format	Analyzed
Experimental factors	Proteins within SDS-PAGE bands were digested with trypsin
Experimental features	Protein identification using MASCOT 2.4.0 engine against a non-redundant SwissProt database with Animals taxonomy restriction
Data source location	Laboratory of Proteomics and Mass Spectrometry, Malopolska Centre of Biotechnology, Jagiellonian University, Krakow, Poland
*Data accessibility*	Data is available within this article

**Value of the data**

• The data provided evidence that lysenin and lysenin-related protein 2 (LRP-2, fetidin) are present in earthworms from both *Eisenia andrei* and *E. fetida* species. They are accompanied by LRP-3 and LRP-1 in most specimens of the former and latter species, respectively. These inter-species differences are worth of further elucidation.
• The data showed the presence of numerous modifications of the unique peptide sequences of lysenin family proteins. From that three-dimensional models can be obtained, useful for comparative studies on modifications of domain organization.
• Exploiting the present data can contribute to elucidate interactions of lysenin and particular LRPs with sphingomyelin and other target molecules.

## 1 Data

The present data consist of detailed characteristics of proteins from lysenin family present in coelomocytes of lumbricid earthworms *Eisenia andrei* and *E. fetida*, the latter without and with the MUG-like fluorophore (RfM- and EfM+, respectively), identified as described in [1].

## 2 Experimental design, materials and methods

Table 1 shows sequence similarity of lysenin (L) and lysenin related proteins 1–3 (LRP-1, LRP-2/fetidin, LRP-3), their molecular weights and accession numbers (top) and the result of (uniprot) sequence alignment (bottom).

Proteins from lysenin family were detected by Western blotting (WB) in coelomocyte-containing coelomic fluid of 9 adult earthworms, i.e. the 3 specimens (1–3) from the each group of earthworms, Ea, EfM−, EfM+, as described in [1]. The chemiluminescent signals were observed at the molecular weights 35–39 kDa; single (in Ea1) or double (in all remaining samples) WB bands were noticeable in all *E. andrei* and *E. fetida* specimens (see Fig. 3A in [1]). SDS-PAGE bands corresponding to molecular weight of WB bands (see Fig. 3B in [1]) were subjected to LC-MS/MS analysis.

### 2.1 Protein identification by LC-MS/MS analysis

#### 2.1.1 Sample preparation procedure prior to LC-MS/MS analysis

Peptide sample preparation for LC-MS/MS analysis was performed as described in Mikula et al. [2]. Briefly, the excised gel pieces were destained at 37 °C by washing several times in 25% and 50% acetonitrile in 25 mM ammonium bicarbonate buffer, reduced with 50 mM DTT at 37 °C for 45 min and alkylated with 55 mM iodoacetamide for 2 h at room temperature in the dark. Excess reagents were washed out with 50% acetonitrile in 25 mM ammonium bicarbonate. Gel pieces were dehydrated in 100% acetonitrile, dried and rehydrated in 15 μl of Sequencing Grade Modified Trypsin solution (10 ng/μl in 25 mM NH_4_HCO_3_, pH 8.0, Promega). After that, additional 25 μl of 25 mM NH_4_HCO_3_ was added. The digestion was carried out at 37 °C overnight. Tryptic peptides were extracted from gel plugs, dried and resuspended in 2% acetronitrile with 0.05% trifluoroacetic acid (TFA).

#### 2.1.2 LC-MS/MS measurements

The LC-MS/MS measurements of peptide solutions were carried out on Q-Exactive mass spectrometer (Thermo Fisher Scientific, Bremen, Germany) equipped with a DPV-550 Digital PicoView nanospray source and connected to an UltiMate 3000RS LC nanoSystem (Dionex). Samples were injected on a C18 precolumn (Acclaim PepMap Nano trap Column) using 2% acetonitrile with 0.05% TFA as a mobile phase, and further separated on a 15 cm×75 μm C18 reversed phase column (Acclaim PepMap 75 μm 100 Å Nano Series TM Column) with gradient from 2% to 40% ACN in 0.05% formic acid for 30 min at a flow rate of 300 nl/min.

The Q-Exactive “sensitive method” was applied based on Kelstrup et al. [3] with slight modifications. The electrospray voltage was set to 2.2 kV, and the ion transfer tube temperature was 250 °C. Full MS scans were acquired in the Orbitrap mass analyzer over m/z 300–2000 range with resolution 70,000 (at m/z 200). The target value was 1.00E+06. The top six most intense peaks with charge state ≥2 were fragmented in the HCD collision cell normalized collision energy of 27%, (the isolation window was 1.2 m/z).Tandem mass spectrum was acquired in the Orbitrap mass analyzer with resolution 35,000 at m/z 200. The target value was 5.00E+05. The ion selection threshold was 1.00E+05 counts, and the maximum allowed ion accumulation times were 120 ms for full MS scans and 120 ms for tandem mass spectrum, dynamic exclusion was set to 30 s.

#### 2.1.3 LC-MS/MS data analysis

Database searching of RAW files was performed in Proteome Discoverer 1.4 (Thermo Fisher Scientific). MASCOT 2.4.0 was used for database searching against a non-redundant SwissProt database with Animals taxonomy restriction (release May 2014, 103370 sequences). The following search parameters were applied: up to one missed cleavages allowed for full tryptic digestion, precursor mass tolerance 6 ppm, product ions mass tolerance 0.02 Da, fixed modification: carbamidomethylation (C), variable modifications: oxidation (M), deamidated (NQ) phosphorylation (STY).

The results of LC-MS/MS analysis are shown in Table 2. The proteins listed in Table 2 were identified based on at least one unique peptide (see sequence alignment – lower panel in Table 1). Supplementary Table 1 A, B, and C proves the reliability of the findings and shows quality of unique peptides of lysenin and LRPs in each of 9 specimens of *Eisenia* sp.

## Figures and Tables

**Table 1:** **Top:** Sequence similarity of lysenin (L) and lysenin related proteins 1–3 (LRP-1, LRP-2/fetidin, LRP-3), their molecular weights and accession numbers. **Bottom:** The result of (uniprot) sequence alignment.

**Proteins Accession numbers**	**Molecular weight kDa**	**L=lysenin TXL_EISFO****O18423**	**LRP-1 TXLR1_EISFO****O18424**	**LRP-2/fetidin TXLR2_EISFO****O18425**	**LRP-3 TXLR3_EISFO****Q3LX99**
**L=lysenin**	**33.44**	–	75.3%	88.7%	79.7%
**LRP-1**	**33.91**	75.3%	–	75.3%	86.3%
**LRP-2 /fetidin**	**34.14**	88.7%	75.3%	–	78.3%
**LRP-3**	**33.84**	79.7%	86.3%	78.3%	–

**Table 2:** Characteristics of protein identified by LC-MS/MS analysis of coelomocytes from Ea, EfM+, and EfM− earthworms, 3 individuals (No. 1–3) from each group. Lysenin (L) and lysenin-related proteins (LRP-1, LRP-3, LRP-2).

**Samples**		**U P P E R B A N D**					**L O W E R B A N D**				
**Earthworms**	**Proteins**	**Score**	**% coverage**	**# matches**	**# peptides**	**# unique peptides**	**Score**	**% coverage**	**# matches**	**# peptides**	**# unique peptides**
**Ea1**	**L**	**–**	**–**	**–**	**–**	**–**	**1963**	**21**	**87**	**8**	**2**
	LRP-1	–	–	–	–	–	–	–	–	–	–
	**LRP-2**	**–**	**–**	**–**	**–**	**–**	**1313**	**28**	**59**	**11**	**4**
	**LRP-3**	–	–	–	–	–	–	–	–	–	–
											
**Ea2**	**L**	**8405**	**67**	**370**	**19**	**8**	**2133**	**27**	**90**	**11**	**3**
	LRP-1	–	–	–	–	–	–	–	–	–	–
	**LRP-2**	**4306**	**48**	**189**	**16**	**7**	**4247**	**55**	**187**	**17**	**7**
	**LRP-3**	**–**	**–**	**–**	**–**	**–**	**1196**	**27**	**44**	**9**	**4**
											
**Ea3**	**L**	**3006**	**47**	**127**	**15**	**5**	**7319**	**70**	**325**	**19**	**9**
	LRP-1	–	–	–	–	–	–	–	–	–	–
	**LRP-2**	**4306**	**48**	**189**	**16**	**7**	**4860**	**50**	**204**	**17**	**7**
	**LRP-3**	**1168**	**15**	**300**	**44**	**2**	**–**	**–**	**–**	**–**	**–**
											
**EfM+1**	**L**	**1044**	**37**	**50**	**1**	**2**	**2431**	**21**	**102**	**8**	**2**
	**LRP-1**	**–**	**–**	**–**	**–**	**–**	**89**	**7**	**4**	**4**	**2**
	**LRP-2**	**746**	**25**	**35**	**10**	**2**	**1279**	**50**	**83**	**12**	**4**
	LRP-3	–	–	–	–	–	–	–	–	–	–
											
**EfM+2**	**L**	**1659**	**17**	**69**	**7**	**1**	**2831**	**17**	**121**	**7**	**1**
	**LRP-1**	**–**	**–**	**–**	**–**	**–**	**–**	**–**	**–**	**–**	**–**
	**LRP-2**	**1510**	**32**	**75**	**12**	**5**	**1966**	**25**	**90**	**11**	**4**
	LRP-3	–	–	–	–	–	–	–	–	–	–
											
**EfM+3**	**L**	**1968**	**31**	**69**	**11**	**4**	**4154**	**33**	**154**	**13**	**5**
	**LRP-1**	**473**	**7**	**24**	**4**	**2**	**508**	**7**	**29**	**4**	**2**
	**LRP-2**	**1235**	**25**	**60**	**10**	**2**	**2570**	**35**	**120**	**12**	**4**
	LRP-3	–	–	–	–	–	–	–	–	–	–
											
**EfM-1**	**L**	**1808**	**21**	**83**	**9**	**2**	**2551**	**21**	**102**	**8**	**2**
	**LRP-1**	**–**	**–**	**–**	**–**	**–**	**66**	**7**	**6**	**4**	**2**
	**LRP-2**	**131**	**22**	**63**	**10**	**3**	**1898**	**42**	**89**	**12**	**5**
	LRP-3	–	–	–	–	–	–	–	–	–	–
											
**EfM-2**	**L**	**2290**	**38**	**86**	**13**	**5**	**4882**	**53**	**178**	**15**	**6**
	**LRP-1**	**627**	**7**	**33**	**4**	**2**	**667**	**7**	**34**	**4**	**2**
	**LRP-2**	**1665**	**25**	**81**	**10**	**2**	**3118**	**35**	**133**	**13**	**4**
	LRP-3	–	–	–	–	–	–	–	–	–	–
											
**EfM-3**	**L**	**1997**	**31**	**67**	**11**	**4**	**2995**	**32**	**113**	**12**	**4**
	**LRP-1**	**371**	**7**	**26**	**4**	**2**	**353**	**5**	**20**	**3**	**2**
	**LRP-2**	**1240**	**25**	**49**	**9**	**1**	**1392**	**32**	**60**	**11**	**3**
	LRP-3	–	–	–	–	–	–	–	–	–	–
