# Supplementary material for: Data on proteins of lysenin family in coelomocytes of Eisenia andrei and E. fetida obtained by tandem mass spectrometry coupled with liquid chromatography
Source: Data Brief. 2016 Sep 29;9:629–34. doi: 10.1016/j.dib.2016.09.035 (PMC5066195; doi:10.1016/j.dib.2016.09.035)
Supplement: Supplementary file 1 — Supplementary material [file mmc1.doc]

The author declare that there are no conflicts of interest.

Barbara Płytycz
