# Supplementary material for: Data on proteins of lysenin family in coelomocytes of Eisenia andrei and E. fetida obtained by tandem mass spectrometry coupled with liquid chromatography
Source: Data Brief. 2016 Sep 29;9:629–34. doi: 10.1016/j.dib.2016.09.035 (PMC5066195; doi:10.1016/j.dib.2016.09.035)
Supplement: Supplementary file 2 — Supplementary material [file mmc2.doc]

Supplementary Table 1. Unique peptides of lysenin and lysenin-related proteins LRP-1, LRP-2 and LRP-3 identified in lower and upper bands excised from SDS-PAGE electrophoresis of coelomic fluid from specimens of lumbricid earthworms a) *Eisenia andrei* (Ea); b) *E. fetida* with MUG-like fluorophore (EfM+); c) *E. fetida* without MUG-like fluorophore (EfM-) and their modifications^2^.

^1^PSMs: Peptide Spectrum Matches

^2^Modifications: carbamidomethylation (C), variable modifications: oxidation (M), deamidated (NQ), phosphorylation (STY).

^3^m/z: mass to charge ratio

Supplementary Table 1A. Unique peptides from three specimens of *Eisenia andrei* signed as Ea1, Ea2, Ea3.

| **Earthworm – band**  **Protein**  Sequence | **# PSMs^1^** | **Protein group accession** | **Modifications^2^** | **Ion Score** | **# Missed Cleavages** | **Retention time [min]** | **m/z^3^ [Da]** | **Charge** |
| --- | --- | --- | --- | --- | --- | --- | --- | --- |
| **Ea1 – lower band** |  |  |  |  |  |  |  |  |
| **Lysenin** |  |  |  |  |  |  |  |  |
| VIEHTITIPPTSK | 45 | O18423 |  | 71 | 0 | 21.31 | 718.41107 | 2 |
| GSTSVDQKITITK | 2 | O18423 |  | 56 | 1 | 18.48 | 689.38208 | 2 |
| **LRP-2** |  |  |  |  |  |  |  |  |
| SGLcYYDGPATDVYcLDKR | 5 | O18425 | C4; C15; | 88 | 1 | 25.46 | 1127.00232 | 2 |
| SGLcYYDGPATDVYcLDK | 4 | O18425 | C4; C15; | 76 | 0 | 26.95 | 1048.94873 | 2 |
| AGIAEGYEQIEVDVVAVWK | 4 | O18425 |  | 38 | 0 | 32.46 | 692.69232 | 3 |
| WILEVVKP | 2 | O18425 |  | 27 | 0 | 27.27 | 492.29999 | 2 |
| EDKWILEVVKP | 4 | O18425 |  | 17 | 1 | 27.28 | 678.38184 | 2 |
| **Ea2 – upper band** |  |  |  |  |  |  |  |  |
| **Lysenin** |  |  |  |  |  |  |  |  |
| TLNTmYSGYEYAYSSDQGGIYFDQGTDNPK | 16 | O18423 | M5; | 94 | 0 | 28.23 | 1134.48486 | 3 |
| AAEGYEQIEVDVVAVWK | 18 | O18423 |  | 90 | 0 | 32.30 | 953.48120 | 2 |
| SGLcYDDGPATNVYcLDKR | 10 | O18423 | C4; C15; | 88 | 1 | 24.18 | 1102.48914 | 2 |
| SGLcYDDGPATNVYcLDK | 3 | O18423 | C4; C15; | 87 | 0 | 26.13 | 1024.44019 | 2 |
| SGLcYDDGPATnVYcLDKR | 1 | O18423 | C4; N12; C15; | 77 | 1 | 24.75 | 1102.98682 | 2 |
| VIEHTITIPPTSK | 65 | O18423 |  | 75 | 0 | 21.79 | 718.41101 | 2 |
| TLNTMYSGYEYAYSSDQGGIYFDQGTDNPK | 4 | O18423 |  | 68 | 0 | 29.16 | 847.11761 | 4 |
| FVLYEDWGGFR | 18 | O18423 |  | 67 | 0 | 29.74 | 694.83466 | 2 |
| TLnTmYSGYEYAYSSDqGGIYFDQGTDNPK | 5 | O18423 | N3; M5; Q17; | 67 | 0 | 35.06 | 1135.14673 | 3 |
| TLnTMYSGYEYAYSSDQGGIYFDQGTDNPK | 11 | O18423 | N3; | 61 | 0 | 30.51 | 1129.48352 | 3 |
| WQLnADVGGADIEYmYLIDEVTPIGGTQSIPQVITSR | 11 | O18423 | N4; M15; | 58 | 0 | 35.84 | 1356.33838 | 3 |
| SGLcYDDGPATnVYcLDK | 2 | O18423 | C4; N12; C15; | 56 | 0 | 27.20 | 1024.93530 | 2 |
| WQLNADVGGADIEYmYLIDEVTPIGGTQSIPQVITSR | 2 | O18423 | M15; | 47 | 0 | 36.36 | 1356.01123 | 3 |
| TLNTmYSGYEYAYSSDqGGIYFDQGTDNPK | 35 | O18423 | M5; Q17; | 44 | 0 | 42.51 | 1134.81934 | 3 |
| QIILGK | 12 | O18423 |  | 32 | 0 | 22.68 | 336.22610 | 2 |
| WILEVVG | 1 | O18423 |  | 27 | 0 | 31.72 | 408.23602 | 2 |
| WqLnADVGGADIEYmYLIDEVTPIGGTQSIPQVITSR | 1 | O18423 | Q2; N4; M15; | 24 | 0 | 37.17 | 1017.75366 | 4 |
| AAEGYEqIEVDVVAVWK | 1 | O18423 | Q7; | 18 | 0 | 42.30 | 953.97797 | 2 |
| TLnTMYSGYEYAYSSDqGGIYFDQGTDNPK | 1 | O18423 | N3; Q17; | 15 | 0 | 33.84 | 1129.81372 | 3 |
| SGLcyDDGPATnVYcLDKR | 4 | O18423 | C4; Y5; N12; C15; | 14 | 1 | 24.00 | 762.30988 | 3 |
| EDKWILEVVG | 5 | O18423 |  | 13 | 1 | 32.11 | 594.31665 | 2 |
| **LRP-2** |  |  |  |  |  |  |  |  |
| SGLcYYDGPATDVYcLDKR | 5 | O18425 | C4; C15; | 103 | 1 | 25.04 | 1127.00061 | 2 |
| AGIAEGYEQIEVDVVAVWK | 2 | O18425 |  | 102 | 0 | 33.00 | 1038.53735 | 2 |
| EIEHTITIPPTSK | 12 | O18425 |  | 76 | 0 | 22.97 | 733.39819 | 2 |
| SGLcYYDGPATDVYcLDK | 3 | O18425 | C4; C15; | 71 | 0 | 27.51 | 1048.94983 | 2 |
| FVLYEDmYGFR | 21 | O18425 | M7; | 69 | 0 | 28.25 | 728.33368 | 2 |
| TLNTmYSGYEYAYSSDQGGIYFDQGSDNPK | 3 | O18425 | M5; | 62 | 0 | 28.12 | 1129.81482 | 3 |
| EIYLGETEIR | 4 | O18425 |  | 57 | 0 | 25.63 | 611.81873 | 2 |
| FVLYEDMYGFR | 1 | O18425 |  | 47 | 0 | 29.70 | 720.33624 | 2 |
| TLNTMYSGYEYAYSSDqGGIYFDQGSDNPK | 4 | O18425 | Q17; | 44 | 0 | 28.80 | 1124.81396 | 3 |
| TLnTmYSGYEYAYSSDqGGIYFDQGSDNPK | 1 | O18425 | N3; M5; Q17; | 44 | 0 | 27.67 | 1695.21082 | 2 |
| TLNTmYSGYEYAYSSDqGGIYFDQGSDNPK | 3 | O18425 | M5; Q17; | 41 | 0 | 31.30 | 1130.14648 | 3 |
| WILEVVKP | 2 | O18425 |  | 31 | 0 | 27.33 | 492.29984 | 2 |
| EDKWILEVVKP | 4 | O18425 |  | 21 | 1 | 27.32 | 678.38153 | 2 |
| AGIAEGYEqIEVDVVAVWK | 1 | O18425 | Q9; | 14 | 0 | 33.57 | 1039.03345 | 2 |
| **Ea2 – lower band** |  |  |  |  |  |  |  |  |
| **Lysenin** |  |  |  |  |  |  |  |  |
| VIEHTITIPPTSK | 5 | O18423 |  | 71 | 0 | 21.01 | 718.40918 | 2 |
| FVLYEDWGGFR | 2 | O18423 |  | 54 | 0 | 29.89 | 694.83240 | 2 |
| QIILGK | 1 | O18423 |  | 30 | 0 | 19.58 | 336.22546 | 2 |
| **LRP-2** |  |  |  |  |  |  |  |  |
| SGLcYYDGPATDVYcLDKR | 8 | O18425 | C4; C15; | 95 | 1 | 25.62 | 1126.99695 | 2 |
| SGLcYYDGPATDVYcLDK | 3 | O18425 | C4; C15; | 84 | 0 | 27.55 | 1048.94666 | 2 |
| TLNTmYSGYEYAYSSDQGGIYFDQGSDNPK | 5 | O18425 | M5; | 73 | 0 | 28.81 | 1129.80884 | 3 |
| FVLYEDmYGFR | 24 | O18425 | M7; | 72 | 0 | 28.32 | 728.33203 | 2 |
| EIEHTITIPPTSK | 24 | O18425 |  | 70 | 0 | 20.97 | 733.39496 | 2 |
| TLnTmYSGYEYAYSSDQGGIYFDQGSDNPK | 17 | O18425 | N3; M5; | 62 | 0 | 29.37 | 1130.14270 | 3 |
| EIYLGETEIR | 8 | O18425 |  | 61 | 0 | 26.19 | 611.81714 | 2 |
| TLnTmYSGYEYAYSSDqGGIYFDQGSDNPK | 1 | O18425 | N3; M5; Q17; | 31 | 0 | 27.69 | 1695.21033 | 2 |
| WILEVVKP | 8 | O18425 |  | 29 | 0 | 27.45 | 492.29822 | 2 |
| EDKWILEVVKP | 6 | O18425 |  | 23 | 1 | 27.43 | 678.38043 | 2 |
| AGIAEGYEQIEVDVVAVWK | 1 | O18425 |  | 18 | 0 | 32.61 | 1038.53369 | 2 |
| **LRP-3** |  |  |  |  |  |  |  |  |
| FVLFEDSSGTR | 1 | Q3LX99 |  | 75 | 0 | 26.26 | 629.30634 | 2 |
| QIHLGTTAVR | 2 | Q3LX99 |  | 54 | 0 | 18.22 | 365.87909 | 3 |
| TLTATHTVGR | 2 | Q3LX99 |  | 50 | 0 | 16.02 | 528.79236 | 2 |
| QEYmTVIER | 1 | Q3LX99 | M4; | 40 | 0 | 19.85 | 592.78271 | 2 |
| SNLFK | 1 | Q3LX99 |  | 18 | 0 | 17.51 | 304.67279 | 2 |
| EDKWILEVVNP | 1 | Q3LX99 |  | 15 | 1 | 30.20 | 671.35480 | 2 |
| **Ea3 – upper band** |  |  |  |  |  |  |  |  |
| **Lysenin** |  |  |  |  |  |  |  |  |
| SGLcYDDGPATNVYcLDK | 1 | O18423 | C4; C15; | 80 | 0 | 25.62 | 1024.43848 | 2 |
| VIEHTITIPPTSK | 20 | O18423 |  | 71 | 0 | 20.27 | 718.41119 | 2 |
| SGLcYDDGPATNVYcLDKR | 4 | O18423 | C4; C15; | 68 | 1 | 24.15 | 735.32892 | 3 |
| FVLYEDWGGFR | 6 | O18423 |  | 63 | 0 | 30.28 | 694.83453 | 2 |
| TLNTmYSGYEYAYSSDQGGIYFDQGTDNPK | 1 | O18423 | M5; | 59 | 0 | 27.71 | 1701.22546 | 2 |
| SGLcYDDGPATnVYcLDK | 1 | O18423 | C4; N12; C15; | 39 | 0 | 27.16 | 1024.93140 | 2 |
| QIILGK | 5 | O18423 |  | 32 | 0 | 18.87 | 336.22589 | 2 |
| TLNTmYSGYEYAYSSDqGGIYFDQGTDNPK | 3 | O18423 | M5; Q17; | 24 | 0 | 27.73 | 851.36493 | 4 |
| EDKWILEVVG | 1 | O18423 |  | 17 | 1 | 29.99 | 594.31836 | 2 |
| **LRP-2** |  |  |  |  |  |  |  |  |
| SGLcYYDGPATDVYcLDKR | 7 | O18425 | C4; C15; | 105 | 1 | 25.00 | 1127.00073 | 2 |
| SGLcYYDGPATDVYcLDK | 4 | O18425 | C4; C15; | 92 | 0 | 26.98 | 1048.94836 | 2 |
| TLNTmYSGYEYAYSSDQGGIYFDQGSDNPK | 3 | O18425 | M5; | 80 | 0 | 28.05 | 1129.81055 | 3 |
| FVLYEDmYGFR | 25 | O18425 | M7; | 72 | 0 | 29.15 | 728.33331 | 2 |
| EIEHTITIPPTSK | 27 | O18425 |  | 70 | 0 | 21.35 | 733.39758 | 2 |
| TLNTmYSGYEYAYSSDqGGIYFDQGSDNPK | 12 | O18425 | M5; Q17; | 63 | 0 | 29.10 | 1130.14832 | 3 |
| TLnTmYSGYEYAYSSDqGGIYFDQGSDNPK | 3 | O18425 | N3; M5; Q17; | 52 | 0 | 29.61 | 1130.47559 | 3 |
| EIYLGETEIR | 8 | O18425 |  | 37 | 0 | 28.86 | 611.81830 | 2 |
| WILEVVKP | 10 | O18425 |  | 31 | 0 | 27.83 | 492.29956 | 2 |
| EDKWILEVVKP | 5 | O18425 |  | 20 | 1 | 27.33 | 678.38086 | 2 |
| **LRP-3** |  |  |  |  |  |  |  |  |
| FVLFEDSSGTR | 1 | Q3LX99 |  | 72 | 0 | 26.12 | 629.30756 | 2 |
| TLTATHTVGR | 2 | Q3LX99 |  | 50 | 0 | 15.88 | 528.79291 | 2 |
| **Ea3 – lower band** |  |  |  |  |  |  |  |  |
| **Lysenin** |  |  |  |  |  |  |  |  |
| SGLcYDDGPATNVYcLDKR | 6 | O18423 | C4; C15; | 104 | 1 | 23.57 | 1102.49365 | 2 |
| SGLcYDDGPATnVYcLDKR | 4 | O18423 | C4; N12; C15; | 103 | 1 | 24.61 | 1102.98376 | 2 |
| AAEGYEQIEVDVVAVWK | 8 | O18423 |  | 102 | 0 | 30.76 | 953.48248 | 2 |
| SGLcYDDGPATNVYcLDK | 3 | O18423 | C4; C15; | 84 | 0 | 26.01 | 1024.43774 | 2 |
| VIEHTITIPPTSK | 63 | O18423 |  | 83 | 0 | 21.71 | 718.41119 | 2 |
| TLNTmYSGYEYAYSSDQGGIYFDQGTDNPK | 13 | O18423 | M5; | 72 | 0 | 29.75 | 1134.48047 | 3 |
| SGLcYDDGPATnVYcLDK | 1 | O18423 | C4; N12; C15; | 68 | 0 | 27.06 | 1024.93420 | 2 |
| FVLYEDWGGFR | 13 | O18423 |  | 67 | 0 | 32.54 | 694.83484 | 2 |
| WQLNADVGGADIEYmYLIDEVTPIGGTQSIPQVITSR | 1 | O18423 | M15; | 44 | 0 | 36.13 | 1017.25482 | 4 |
| TLnTmYSGYEYAYSSDQGGIYFDQGTDNPK | 42 | O18423 | N3; M5; | 43 | 0 | 34.84 | 1134.81311 | 3 |
| WqLNADVGGADIEYmYLIDEVTPIGGTQSIPQVITSR | 7 | O18423 | Q2;M15; | 40 | 0 | 35.78 | 1356.34326 | 3 |
| ITITKGmK | 1 | O18423 | M7; | 36 | 1 | 15.22 | 454.26727 | 2 |
| QIILGK | 14 | O18423 |  | 32 | 0 | 23.70 | 336.22577 | 2 |
| qIILGK | 1 | O18423 | Q1; | 23 | 0 | 20.12 | 336.71741 | 2 |
| TLNTMYSGYEYAYSSDqGGIYFDQGTDNPK | 2 | O18423 | Q17; | 16 | 0 | 29.08 | 1129.48584 | 3 |
| WqLnADVGGADIEYmYLIDEVTPIGGTqSIPQVITSR | 2 | O18423 | Q2; N4; M15; Q28; | 13 | 0 | 36.71 | 1356.99573 | 3 |
| EDKWILEVVG | 6 | O18423 |  | 13 | 1 | 31.95 | 594.31757 | 2 |
| **LRP-2** |  |  |  |  |  |  |  |  |
| SGLcYYDGPATDVYcLDKR | 5 | O18425 | C4; C15; | 85 | 1 | 25.89 | 751.66895 | 3 |
| TLnTmYSGYEYAYSSDQGGIYFDQGSDNPK | 10 | O18425 | N3; M5; | 83 | 0 | 28.55 | 1130.14514 | 3 |
| TLNTmYSGYEYAYSSDQGGIYFDQGSDNPK | 3 | O18425 | M5; | 77 | 0 | 28.05 | 1129.81628 | 3 |
| SGLcYYDGPATDVYcLDK | 3 | O18425 | C4; C15; | 75 | 0 | 26.92 | 1048.94873 | 2 |
| EIEHTITIPPTSK | 14 | O18425 |  | 73 | 0 | 22.82 | 733.39838 | 2 |
| FVLYEDmYGFR | 18 | O18425 | M7; | 70 | 0 | 27.68 | 728.33325 | 2 |
| EIYLGETEIR | 4 | O18425 |  | 59 | 0 | 25.49 | 611.81842 | 2 |
| WILEVVKP | 3 | O18425 |  | 23 | 0 | 27.24 | 492.29959 | 2 |
| EDKWILEVVKP | 6 | O18425 |  | 21 | 1 | 27.26 | 678.38147 | 2 |

Supplementary Table 1B. Unique peptides from three specimens of *Eisenia fetida* MUG-like-positive (EfM+) designed as EfM+1, EfM+2, EfM+3.

| **Earthworm – band**  **Protein**  Sequence | **# PSMs^1^** | **Protein group accession** | **Modifications^2^** | **Ion Score** | **#Missed Cleavages** | **Retention time**  **[min]** | **m/z^3^**  **[Da]** | **Charge** |
| --- | --- | --- | --- | --- | --- | --- | --- | --- |
| **EfM+1 – upper band** |  |  |  |  |  |  |  |  |
| **Lysenin** |  |  |  |  |  |  |  |  |
| VIEHTITIPPTSK | 14 | O18423 |  | 72 | 0 | 21.52 | 718.41132 | 2 |
| GSTSVDQKITITK | 2 | O18423 |  | 63 | 1 | 18.40 | 689.38116 | 2 |
| WQLnADVGGADIEYmYLIDEVTPIGGTQSIPQVITSR | 3 | O18423 | N4; M15; | 23 | 0 | 35.88 | 1017.50916 | 4 |
| **LRP-2** |  |  |  |  |  |  |  |  |
| SGLcYYDGPATDVYcLDKR | 2 | O18425 | C4; C15; | 77 | 1 | 24.97 | 751.66956 | 3 |
| SGLcYYDGPATDVYcLDK | 1 | O18425 | C4; C15; | 63 | 0 | 27.05 | 1048.94934 | 2 |
| EDKWILEVVKP | 1 | O18425 |  | 15 | 1 | 26.83 | 452.59109 | 3 |
| **EfM+1 – lower band** |  |  |  |  |  |  |  |  |
| **Lysenin** |  |  |  |  |  |  |  |  |
| VIEHTITIPPTSK | 62 | O18423 |  | 71 | 0 | 22.99 | 718.41125 | 2 |
| GSTSVDQKITITK | 2 | O18423 |  | 67 | 1 | 18.34 | 459.92300 | 3 |
| **LRP-2** |  |  |  |  |  |  |  |  |
| SGLcYYDGPATDVYcLDKR | 7 | O18425 | C4; C15; | 85 | 1 | 25.82 | 751.66992 | 3 |
| AGIAEGYEQIEVDVVAVWK | 3 | O18425 |  | 65 | 0 | 32.95 | 1038.53674 | 2 |
| TLNTMYSGYEYAYSSDQGGIYFDQGSDNPK | 1 | O18425 |  | 34 | 0 | 26.85 | 1124.47717 | 3 |
| WILEVVKP | 2 | O18425 |  | 24 | 0 | 27.18 | 492.29993 | 2 |
| EDKWILEVVKP | 4 | O18425 |  | 22 | 1 | 27.21 | 678.38245 | 2 |
| TLnTMYsGYEYAYSSDQGGIYFDQGSDNPK | 10 | O18425 | N3; S7; | 18 | 0 | 29.28 | 1151.47095 | 3 |
| TLNTmYsGYEYAYSSDQGGIYFDQGSDNPK | 8 | O18425 | M5; S7; | 18 | 0 | 29.24 | 1156.46362 | 3 |
| WqLNADVGGADIEYMYLIDEVTPIGGtLSIPQVIK | 10 | O18425 | Q2; T27; | 10 | 0 | 34.81 | 1300.64429 | 3 |
| **LRP-1** |  |  |  |  |  |  |  |  |
| EYmTVISR | 1 | O18424 | M3; | 52 | 0 | 19.24 | 507.75003 | 2 |
| KEYmTVISR | 1 | O18424 | M4; | 39 | 1 | 17.07 | 571.79712 | 2 |
| **EfM+2 – upper band** |  |  |  |  |  |  |  |  |
| **Lysenin** |  |  |  |  |  |  |  |  |
| VIEHTITIPPTSK | 16 | O18423 |  | 69 | 0 | 20.91 | 718.40930 | 2 |
| **LRP-2** |  |  |  |  |  |  |  |  |
| SGLcYYDGPATDVYcLDKR | 1 | O18425 | C4; C15; | 59 | 1 | 24.95 | 751.66693 | 3 |
| FVLYEDmYGFR | 1 | O18425 | M7; | 56 | 0 | 27.30 | 728.33344 | 2 |
| SGLcYYDGPATDVYcLDK | 2 | O18425 | C4; C15; | 51 | 0 | 28.61 | 1048.95483 | 2 |
| AGIAEGYEQIEVDVVAVWK | 1 | O18425 |  | 36 | 0 | 32.64 | 692.69147 | 3 |
| WILEVVKP | 13 | O18425 |  | 32 | 0 | 27.77 | 492.29834 | 2 |
| EDKWILEVVKP | 4 | O18425 |  | 17 | 1 | 27.32 | 678.38080 | 2 |
| **EfM+2 – lower band** |  |  |  |  |  |  |  |  |
| **Lysenin** |  |  |  |  |  |  |  |  |
| VIEHTITIPPTSK | 62 | O18423 |  | 74 | 0 | 24.09 | 718.40912 | 2 |
| **LRP-2** |  |  |  |  |  |  |  |  |
| SGLcYYDGPATDVYcLDKR | 14 | O18425 | C4; C15; | 90 | 1 | 25.20 | 1126.99719 | 2 |
| SGLcYYDGPATDVYcLDK | 4 | O18425 | C4; C15; | 84 | 0 | 26.89 | 1048.94666 | 2 |
| EIYLGETEIR | 1 | O18425 |  | 55 | 0 | 24.84 | 611.81842 | 2 |
| WILEVVKP | 8 | O18425 |  | 31 | 0 | 28.28 | 492.29883 | 2 |
| EDKWILEVVKP | 4 | O18425 |  | 21 | 1 | 27.31 | 452.58929 | 3 |
| **EfM+3 – upper band** |  |  |  |  |  |  |  |  |
| **Lysenin** |  |  |  |  |  |  |  |  |
| SGLcYDDGPATNVYcLDKR | 5 | O18423 | C4; C15; | 96 | 1 | 24.17 | 1102.48877 | 2 |
| SGLcYDDGPATNVYcLDK | 2 | O18423 | C4; C15; | 92 | 0 | 26.12 | 1024.43579 | 2 |
| SGLcYDDGPATnVYcLDK | 1 | O18423 | C4; N12; C15; | 83 | 0 | 27.20 | 1024.93433 | 2 |
| VIEHTITIPPTSK | 11 | O18423 |  | 66 | 0 | 20.43 | 718.41162 | 2 |
| GSTSVDQKITITK | 2 | O18423 |  | 61 | 1 | 18.49 | 689.38159 | 2 |
| SGLcYDDGPATnVYcLDKR | 1 | O18423 | C4;N12; C15; | 54 | 1 | 24.92 | 1102.98633 | 2 |
| **LRP-2** |  |  |  |  |  |  |  |  |
| SGLcYYDGPATDVYcLDKR | 1 | O18425 | C4; C15; | 84 | 1 | 25.04 | 751.66608 | 3 |
| WILEVVKP | 8 | O18425 |  | 32 | 0 | 28.37 | 492.29968 | 2 |
| EDKWILEVVKP | 4 | O18425 |  | 21 | 1 | 27.40 | 678.38159 | 2 |
| **LRP-1** |  |  |  |  |  |  |  |  |
| KEYmTVISR | 8 | O18424 | M4; | 58 | 1 | 17.38 | 571.79724 | 2 |
| EYmTVISR | 8 | O18424 | M3; | 51 | 0 | 22.80 | 507.75278 | 2 |
| KEYMTVISR | 3 | O18424 |  | 47 | 1 | 18.93 | 376.20261 | 3 |
| EYMTVISR | 1 | O18424 |  | 45 | 0 | 22.09 | 499.75262 | 2 |
| **EfM+3 – lower band** |  |  |  |  |  |  |  |  |
| **Lysenin** |  |  |  |  |  |  |  |  |
| SGLcYDDGPATNVYcLDKR | 4 | O18423 | C4; C15; | 85 | 1 | 24.19 | 1102.49011 | 2 |
| GSTSVDQKITITK | 2 | O18423 |  | 76 | 1 | 18.54 | 459.92358 | 3 |
| VIEHTITIPPTSK | 68 | O18423 |  | 73 | 0 | 20.30 | 718.41003 | 2 |
| SGLcYDDGPATnVYcLDKR | 1 | O18423 | C4; N12; C15; | 71 | 1 | 24.71 | 735.66144 | 3 |
| SGLcYDDGPATNVYcLDK | 2 | O18423 | C4; C15; | 67 | 0 | 26.32 | 1024.43811 | 2 |
| ITITKGmK | 1 | O18423 | M7; | 44 | 1 | 15.27 | 454.26706 | 2 |
| SGLcYDDGPATnVYcLDK | 1 | O18423 | C4; N12; C15; | 34 | 0 | 27.13 | 1024.93494 | 2 |
| VIEHTITIPPTSKFTR | 1 | O18423 |  | 11 | 1 | 21.55 | 614.01575 | 3 |
| **LRP-2** |  |  |  |  |  |  |  |  |
| SGLcYYDGPATDVYcLDKR | 13 | O18425 | C4;C15; | 87 | 1 | 25.35 | 1127.00085 | 2 |
| SGLcYYDGPATDVYcLDK | 2 | O18425 | C4; C15; | 73 | 0 | 27.23 | 1048.94971 | 2 |
| TLNTMySGYEYAYSSDQGGIYFDQGSDNPK | 10 | O18425 | Y6; | 36 | 0 | 29.16 | 863.60480 | 4 |
| WILEVVKP | 12 | O18425 |  | 34 | 0 | 28.82 | 492.29974 | 2 |
| EDKWILEVVKP | 9 | O18425 |  | 18 | 1 | 27.81 | 678.38147 | 2 |
| **LRP-1** |  |  |  |  |  |  |  |  |
| KEYmTVISR | 10 | O18424 | M4; | 62 | 1 | 17.13 | 571.79700 | 2 |
| EYMTVISR | 5 | O18424 |  | 38 | 0 | 22.79 | 499.75220 | 2 |
| KEYMTVISR | 5 | O18424 |  | 36 | 1 | 19.43 | 376.20245 | 3 |
| EYmTVISR | 7 | O18424 | M3; | 35 | 0 | 19.33 | 507.75067 | 2 |

Supplementary Table 1C. Unique peptides from three specimens of earthworms *Eisenia fetida* MUG-like–negative (EfM-) designed as EfM-1, EfM-2, EfM-3.

| **Earthworm – band**  **Protein**  Sequence | **# PSMs^1^** | **Protein group accession** | **Modifications^2^** | **Ion Score** | **#Missed Cleavages** | **Retention time**  **[min]** | **m/z^3^**  **[Da]** | **Charge** |
| --- | --- | --- | --- | --- | --- | --- | --- | --- |
| **EfM-1 – upper band** |  |  |  |  |  |  |  |  |
| **Lysenin** |  |  |  |  |  |  |  |  |
| GSTSVDQKITITK | 2 | O18423 |  | 80 | 1 | 18.35 | 689.38171 | 2 |
| VIEHTITIPPTSK | 27 | O18423 |  | 70 | 0 | 21.94 | 718.41345 | 2 |
| EGYVYEnRGSTSVDQK | 1 | O18423 | N7; | 10 | 1 | 19.17 | 916.91815 | 2 |
| **LRP-2** |  |  |  |  |  |  |  |  |
| SGLcYYDGPATDVYcLDKR | 3 | O18425 | C4; C15; | 85 | 1 | 24.96 | 751.66882 | 3 |
| SGLcYYDGPATDVYcLDK | 1 | O18425 | C4; C15; | 40 | 0 | 27.77 | 1048.95203 | 2 |
| WILEVVKP | 2 | O18425 |  | 28 | 0 | 27.27 | 492.29990 | 2 |
| EDKWILEVVKP | 4 | O18425 |  | 16 | 1 | 27.35 | 678.38300 | 2 |
| **EfM-1 – lower band** |  |  |  |  |  |  |  |  |
| **Lysenin** |  |  |  |  |  |  |  |  |
| GSTSVDQKITITK | 2 | O18423 |  | 80 | 1 | 18.51 | 689.38147 | 2 |
| VIEHTITIPPTSK | 42 | O18423 |  | 75 | 0 | 24.41 | 718.41071 | 2 |
| **LRP-2** |  |  |  |  |  |  |  |  |
| SGLcYYDGPATDVYcLDKR | 9 | O18425 | C4; C15; | 79 | 1 | 25.47 | 751.66852 | 3 |
| SGLcYYDGPATDVYcLDK | 3 | O18425 | C4; C15; | 71 | 0 | 27.50 | 1048.94946 | 2 |
| AGIAEGYEQIEVDVVAVWK | 2 | O18425 |  | 58 | 0 | 32.73 | 692.69214 | 3 |
| EIYLGETEIR | 1 | O18425 |  | 55 | 0 | 24.78 | 611.81921 | 2 |
| EDKWILEVVKP | 6 | O18425 |  | 25 | 1 | 27.88 | 678.38147 | 2 |
| TLNTMYSGYEyAYSSDQGGIYFDQGSDNPK | 10 | O18425 | Y11; | 22 | 0 | 29.24 | 1151.13928 | 3 |
| **LRP-1** |  |  |  |  |  |  |  |  |
| KEYmTVISR | 2 | O18424 | M4; | 40 | 1 | 17.14 | 571.79675 | 2 |
| EYmTVISR | 1 | O18424 | M3; | 37 | 0 | 19.40 | 507.74988 | 2 |
| KEYMTVISR | 1 | O18424 |  | 19 | 1 | 19.05 | 563.79974 | 2 |
| **EfM-2 – upper band** |  |  |  |  |  |  |  |  |
| **Lysenin** |  |  |  |  |  |  |  |  |
| SGLcYDDGPATNVYcLDKR | 4 | O18423 | C4; C15; | 106 | 1 | 24.21 | 1102.49036 | 2 |
| SGLcYDDGPATNVYcLDK | 1 | O18423 | C4; C15; | 88 | 0 | 26.09 | 1024.43628 | 2 |
| SGLcYDDGPATnVYcLDKR | 2 | O18423 | C4; N12; C15; | 78 | 1 | 24.73 | 1102.98584 | 2 |
| VIEHTITIPPTSK | 8 | O18423 |  | 70 | 0 | 21.52 | 718.41138 | 2 |
| GSTSVDQKITITK | 2 | O18423 |  | 58 | 1 | 18.49 | 689.38202 | 2 |
| SGLcYDDGPATnVYcLDK | 1 | O18423 | C4; N12; C15; | 39 | 0 | 27.14 | 1024.93481 | 2 |
| SAKAAEGYEqIEVDVVAVWK | 1 | O18423 | Q10; | 30 | 1 | 36.08 | 731.70422 | 3 |
| ITITKGmK | 1 | O18423 | M7; | 27 | 1 | 15.27 | 454.26706 | 2 |
| sGLcYDDGPATNVYcLDKR | 4 | O18423 | S1; C4; C15; | 24 | 1 | 23.97 | 761.98132 | 3 |
| **LRP-2** |  |  |  |  |  |  |  |  |
| SGLcYYDGPATDVYcLDK | 1 | O18425 | C4; C15; | 74 | 0 | 27.28 | 1048.95508 | 2 |
| WILEVVKP | 12 | O18425 |  | 31 | 0 | 28.29 | 492.29977 | 2 |
| EDKWILEVVKP | 6 | O18425 |  | 22 | 1 | 27.84 | 678.38220 | 2 |
| **LRP-1** |  |  |  |  |  |  |  |  |
| KEYmTVISR | 10 | O18424 | M4; | 60 | 1 | 17.53 | 571.79700 | 2 |
| EYmTVISR | 9 | O18424 | M3; | 48 | 0 | 20.38 | 507.74918 | 2 |
| EYMTVISR | 5 | O18424 |  | 42 | 0 | 22.83 | 499.75214 | 2 |
| KEYMTVISR | 5 | O18424 |  | 41 | 1 | 19.44 | 376.20233 | 3 |
| **EfM-2 – lower** |  |  |  |  |  |  |  |  |
| **Lysenin** |  |  |  |  |  |  |  |  |
| SGLcYDDGPATNVYcLDKR | 6 | O18423 | C4; C15; | 104 | 1 | 23.70 | 1102.48987 | 2 |
| SGLcYDDGPATNVYcLDK | 1 | O18423 | C4; C15; | 83 | 0 | 25.67 | 1024.43738 | 2 |
| VIEHTITIPPTSK | 71 | O18423 |  | 77 | 0 | 26.25 | 718.40979 | 2 |
| GSTSVDQKITITK | 2 | O18423 |  | 76 | 1 | 18.40 | 459.92383 | 3 |
| SGLcYDDGPATnVYcLDKR | 1 | O18423 | C4; N12; C15; | 61 | 1 | 24.78 | 1102.98047 | 2 |
| AAEGYEQIEVDVVAVWK | 4 | O18423 |  | 45 | 0 | 30.83 | 953.47913 | 2 |
| WqLNADVGGADIEYmYLIDEVTPIGGTQSIPQVITSR | 3 | O18423 | Q2; M15; | 34 | 0 | 35.97 | 1356.33179 | 3 |
| SGLcYDDGPATnVYcLDK | 1 | O18423 | C4; N12; C15; | 31 | 0 | 27.21 | 1024.93555 | 2 |
| VIEHTITIPPTSKFTR | 1 | O18423 |  | 14 | 1 | 21.50 | 614.01556 | 3 |
| **LRP-2** |  |  |  |  |  |  |  |  |
| AGIAEGYEQIEVDVVAVWK | 10 | O18425 |  | 105 | 0 | 32.50 | 1038.53381 | 2 |
| SGLcYYDGPATDVYcLDKR | 6 | O18425 | C4; C15; | 82 | 1 | 25.35 | 1126.99939 | 2 |
| WILEVVKP | 13 | O18425 |  | 32 | 0 | 27.82 | 1048.95264 | 2 |
| EDKWILEVVKP | 13 | O18425 |  | 21 | 1 | 28.31 | 492.29959 | 2 |
| **LRP-1** |  |  |  |  |  |  |  |  |
| KEYmTVISR | 10 | O18424 | M4; | 60 | 1 | 17.10 | 571.79718 | 2 |
| EYMTVISR | 7 | O18424 |  | 48 | 0 | 24.60 | 499.75336 | 2 |
| KEYMTVISR | 5 | O18424 |  | 41 | 1 | 18.78 | 376.20276 | 3 |
| EYmTVISR | 10 | O18424 | M3; | 39 | 0 | 23.96 | 507.74939 | 2 |
| **Ef M-3 – upper** |  |  |  |  |  |  |  |  |
| **Lysenin** |  |  |  |  |  |  |  |  |
| SGLcYDDGPATNVYcLDK | 2 | O18423 | C4; C15; | 86 | 0 | 25.57 | 1024.43799 | 2 |
| SGLcYDDGPATNVYcLDKR | 4 | O18423 | C4; C15; | 82 | 1 | 23.81 | 735.32764 | 3 |
| GSTSVDQKITITK | 2 | O18423 |  | 78 | 1 | 18.47 | 689.38092 | 2 |
| VIEHTITIPPTSK | 13 | O18423 |  | 71 | 0 | 20.45 | 718.41071 | 2 |
| SGLcYDDGPATnVYcLDKR | 1 | O18423 | C4; N12; C15; | 62 | 1 | 24.83 | 735.65845 | 3 |
| **LRP-2** |  |  |  |  |  |  |  |  |
| SGLcYYDGPATDVYcLDKR | 2 | O18425 | C4; C15; | 83 | 1 | 24.95 | 751.66840 | 3 |
| EDKWILEVVKP | 2 | O18425 |  | 22 | 1 | 27.29 | 678.38165 | 2 |
| **LRP-1** |  |  |  |  |  |  |  |  |
| KEYmTVISR | 8 | O18424 | M4; | 59 | 1 | 17.25 | 571.79712 | 2 |
| EYmTVISR | 8 | O18424 | M3; | 51 | 0 | 19.75 | 507.74973 | 2 |
| KEYMTVISR | 3 | O18424 |  | 41 | 1 | 18.87 | 376.20197 | 3 |
| EYMTVISR | 3 | O18424 |  | 38 | 0 | 21.77 | 499.75189 | 2 |
| **EfM-3 – lower** |  |  |  |  |  |  |  |  |
| **Lysenin** |  |  |  |  |  |  |  |  |
| SGLcYDDGPATNVYcLDKR | 4 | O18423 | C4; C15; | 103 | 1 | 23.66 | 1102.48950 | 2 |
| SGLcYDDGPATNVYcLDK | 1 | O18423 | C4; C15; | 94 | 0 | 25.58 | 1024.43896 | 2 |
| VIEHTITIPPTSK | 56 | O18423 |  | 71 | 0 | 23.83 | 718.41083 | 2 |
| GSTSVDQKITITK | 2 | O18423 |  | 55 | 1 | 18.42 | 689.38153 | 2 |
| ITITKGmK | 1 | O18423 | M7; | 24 | 1 | 15.15 | 454.26712 | 2 |
| SGLcYDDGPATnVYcLDKR | 1 | O18423 | C4; N12; C15; | 18 | 1 | 25.22 | 735.65924 | 3 |
| **LRP-2** |  |  |  |  |  |  |  |  |
| SGLcYYDGPATDVYcLDKR | 2 | O18425 | C4; C15; | 86 | 1 | 25.75 | 751.66968 | 3 |
| AGIAEGYEQIEVDVVAVWK | 3 | O18425 |  | 63 | 0 | 33.01 | 692.69141 | 3 |
| WILEVVKP | 2 | O18425 |  | 23 | 0 | 27.25 | 492.29987 | 2 |
| EDKWILEVVKP | 5 | O18425 |  | 16 | 1 | 27.26 | 452.59012 | 3 |
| **LRP-1** |  |  |  |  |  |  |  |  |
| EYmTVISR | 6 | O18424 | M3; | 71 | 0 | 19.23 | 507.74966 | 2 |
| KEYmTVISR | 8 | O18424 | M4; | 68 | 1 | 17.06 | 571.79681 | 2 |
| EYMTVISR | 2 | O18424 |  | 43 | 0 | 21.73 | 499.75235 | 2 |
| KEYMTVISR | 3 | O18424 |  | 42 | 1 | 18.83 | 563.80029 | 2 |
